# Game On—Complier Average Causal Effect Estimation Reveals Sleeper Effects on Academic Attainment in a Randomized Trial of the Good Behavior Game

**DOI:** 10.1007/s11121-019-01074-6

**Published:** 2020-01-20

**Authors:** Emma Ashworth, Margarita Panayiotou, Neil Humphrey, Alexandra Hennessey

**Affiliations:** 1 Manchester Institute of Education, University of Manchester, Oxford Road, Manchester, UK; 2 School of Psychology, Liverpool John Moores University, Byrom Street, Liverpool, L3 3AF UK

**Keywords:** Good Behavior Game, Academic attainment, Randomized trial, Implementation, Complier average causal effect estimation

## Abstract

To examine the efficacy of the Good Behavior Game (GBG) in improving children’s reading attainment, and the extent to which this varies as a function of cumulative intervention intensity (dosage) and timing of outcome measurement. A 2-year cluster-randomized controlled trial was conducted. Seventy-seven primary schools from three regions in England were randomly assigned to intervention and control groups. Children (*N* = 3084) aged 67 at baseline were the target cohort. The GBG is an interdependent group-contingency behavior management strategy used by teachers in elementary schools. Reading attainment was assessed via national teacher assessment scores at baseline, and the Hodder Group Reading Test at post-test and 1-year post-intervention follow-up. Dosage was assessed using a bespoke online GBG scoreboard system. Multi-level intent-to-treat (ITT) and complier average causal effect (CACE) estimation were utilized. At post-test, no effects of the GBG on children’s reading attainment were found in either the ITT or CACE models. At 1-year follow-up, results remained null in the ITT model, but a significant intervention effect was found among moderate compliers (Δ = 0.10) in the CACE model. The GBG can produce measurable improvements in children’s academic attainment, but these effects may take time to become apparent and are contingent upon implementation dosage falling within an optimal range. The project was supported by funding from the Education Endowment Foundation and the National Institute for Health Research. ISRCTN: 64152096.

**Electronic supplementary material:**

The online version of this article (10.1007/s11121-019-01074-6) contains supplementary material, which is available to authorized users.

The Good Behavior Game (GBG) is an interdependent group-contingency behavior management strategy (Lastrapes 2013), originally developed by Barrish et al. 1969 for use by elementary school teachers. Unsurprisingly, the majority of GBG research to date has focused on behavioral outcomes (Flower et al. 2014). However, the program logic model also predicts improvements in academic outcomes (e.g., literacy, numeracy) in the short-to-medium term (Chan et al. 2012). It is theorized that the GBG facilitates academic progress by socializing children into the role of student, increasing attention and on-task behavior (Ford et al. 2014). Disruptive behaviors are also reduced, improving children’s ability to focus and be more productive (Chan et al. 2012). As a consequence, it is possible that improvements in attainment will only be evidenced in the longer term and not immediately following exposure to the GBG. This is known as a “sleeper effect,” whereby enlarged positive effects are identified at later follow-up, compared to immediately post-intervention (van Aar et al. 2017).

Findings regarding the impact of the GBG on academic attainment, and in particular the point at which such effects might occur, have been equivocal. Regarding immediate impacts, an early study in Baltimore, USA, did not find an impact on reading in 6–8year olds (Dolan et al. 1993). A second trial (Dion et al. 2011) of the GBG combined with peer tutoring only found significant improvements in literacy outcomes in 6–7year olds following exposure to peer tutoring, but *not* the GBG. In contrast, Weis et al. (2015) reported small but significant effects of the GBG on reading and mathematics scores.

Regarding longer-term impacts, later follow-ups have also produced mixed findings. A study utilizing the sample from the first generation of Baltimore trials (from Dolan et al. 1993) found no effects at the intention-to-treat (ITT) level on high school and college outcomes for students who had received the GBG in first and second grade (Hemelt et al. 2013). Conversely, Bradshaw et al.’ (2009) longitudinal study utilizing the sample from the second generation of Baltimore trials reported positive effects on a range of academic outcomes at age 19, after a single year of GBG exposure at age 6–7.

Mixed results notwithstanding, methodological issues in some of the above studies around sampling (e.g., over-representation of inattentive students; Dion et al. 2011), study design (e.g., quasi-experimental; Weis et al. 2015) and the confounding influence of other interventions (as in Bradshaw et al. 2009, who combined the GBG with an intensive enhanced academic curriculum) precluded firm conclusions being drawn, and as such, the extent to which the GBG can improve children’s academic outcomes remains uncertain.

## The (Potential) Importance of Implementation

When evaluating the impact of school-based interventions, it is crucial to consider the way in which they are delivered; levels of adherence to prescribed procedures (*fidelity*) and exposure (*dosage*) have both been shown to be important in this regard (Durlak 2016). However, most studies simply report descriptive data; routine analysis of the moderating role of implementation variability in school-based preventive interventions is still uncommon (Bruhn et al. 2015; Hagermoser Sanetti et al. 2014). Indeed, only a handful of GBG studies have reported implementation data (e.g., Domitrovich et al. 2015; Hagermoser Sanetti and Fallon 2011), and only one has examined its moderating role in student outcomes. Ialongo et al. (1999) found that fidelity moderated the impact of the GBG on reading and mathematics outcomes, with positive outcomes only being identified in high fidelity classrooms. However, this effect varied by gender for reading (i.e., only boys evidenced gains in reading in the high fidelity group) and no main effects analysis was conducted (i.e., analyses were only conducted by gender).

Thus, it is currently unknown whether variability in GBG implementation influences its impact (Berg et al. 2017), and in particular, the moderating role of dosage is yet to be examined. As this variability is inevitable in school-based interventions (Durlak 2016), it is likely that traditional ITT analyses provide a biased estimate of their effects (Jo and Muthén 2001; Peugh et al. 2017). As a supplement to ITT models, complier average causal effect (CACE) estimation offers a robust, unbiased means through which to examine intervention effects while taking levels of implementation into account (Berg et al. 2017). However, it has been given “little to no attention in school psychology” (Peugh and Toland 2017, p. 5). Indeed, only a handful of examples of CACE have been identified in the context of school-based interventions; its application in a *multilevel* context is even rarer (Panayiotou et al. 2019).

## The GBG in England

Previous research into the efficacy of school-based interventions delivered outside their country of origin suggests that cultural transferability may be an issue, with smaller effect sizes sometimes reported when they are “imported” (Wigelsworth et al. 2016). Levels of fit with the new cultural context and local needs can influence their success (Castro et al. 2004). Thus, aspects of the English school system may impact the implementation of the GBG. Teachers in England already struggle with the multiple and competing demands placed upon them; the National Curriculum and priorities set by regulatory boards such as Ofsted also influence the amount of time and support that teachers have to implement additional programs (Illingworth 2007). Furthermore, the perceived social validity (e.g., acceptability, feasibility, utility) of the GBG may influence its implementation. For instance, the prohibition of teacher-student interaction during gameplay sessions has been noted as problematic by some teachers (Chan et al. 2012; Ashworth et al. 2018). The observation culture in English school system also causes anxiety (Illingworth 2007); thus, the coaching element (which involves direct observations) may not be perceived favorably (Ashworth et al. 2018). Such factors are likely to influence teachers’ implementation of the GBG, and the likelihood that said implementation will be sustained (Wehby et al. 2011).

## The Current Study

The GBG was the subject of a successful pilot evaluation in England in 2011–2012 (Chan et al. 2012), with significant improvements reported in a range of behaviors (e.g. attention/concentration). However, this study did not include a control group, limiting the extent to which these improvements could be securely attributed to the GBG, and effects on academic attainment were not examined. Thus, a large cluster randomized controlled trial (RCT) was undertaken to address these issues (Humphrey et al. 2018). Of particular note in the context of the current study are findings from a concurrent paper examining the effects of the GBG on behavioral outcomes. While ITT analyses revealed no impact of the GBG on behavior at immediate post-test, CACE analyses demonstrated large intervention effects among compliers (specifically, reductions in disruptive behavior; Humphrey et al. in press). This finding sets the stage for a scenario in which the aforementioned mechanisms through which GBG is theorized to improve later academic progress are set in motion only in classrooms where a particular threshold of dosage has been met or exceeded.

In light of the preceding literature, the aim of the current study is to improve understanding of the effects of the GBG by examining its impact on children’s reading attainment when tested in isolation, while taking into account implementation variability using multilevel CACE. In order to clarify ambiguity regarding the immediacy of intervention effects, both post-test and 1-year post-intervention follow-up effects are examined.

## Method

### Design

A 2-year cluster-randomized design was utilized (2015–2017), with participating schools as the unit of randomization. The random allocation procedure was conducted independently by a local trials unit. Balance across the arms of the trial in terms of the proportion of children eligible for free school meals (FSMs) and school size was ensured via adaptive stratification. Schools were randomly allocated to one of two arms: (1) GBG (intervention), or (2) usual provision (UP). The trial protocol is available here [https://educationendowmentfoundation.org.uk/projects-and-evaluation/projects/the-good-behaviourgame/]. A mixed-methods implementation and process evaluation (IPE) was also conducted in GBG schools.

Eligible schools were mainstream, state-maintained primary schools in three regions across England. Recruitment occurred between March and July 2015. Participation required consent from the schools’ Head Teachers, child assent, and parental opt-out consent. Sixty-eight parents (2.2%) exercised their right to opt their children out of the trial. The study received approval from the ethics committee of the authors’ host institution.

### Participants

**Fig. 1:**
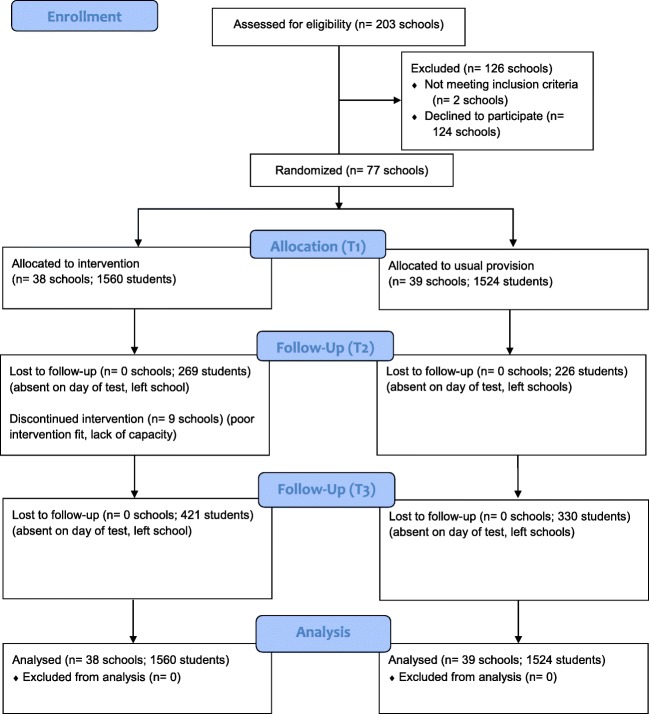
CONSORT flow diagram

The target cohort were *N* = 3084 children aged 67 in 77 mainstream primary schools (see Fig. 1). The composition of participating schools mirrored that of English primary schools regarding size and the proportion of students speaking English as an additional language (EAL), but contained significantly larger proportions of children with special educational needs and disabilities (SEND) and those eligible for FSM, in addition to lower rates of absence and attainment (Table 1). The student sample was also generally above the national average in terms of the proportion of children with an SEND, eligible for FSM, and speaking EAL, while they were below average regarding attainment (DfE 2015). There were no significant differences between trial arms for any of the variables noted above (*F*(7, 68) = 0.78, *p* = .61), indicating good balance and successful randomization.

**Table 1:** Descriptive and demographic data

Dosage	Min–max		Mean		SD	
Games/week 2015/16 (20 weeks total delivery)^†^	0.30–4.45		1.93		1.15	
Games/week 2016/17 (29 weeks total delivery)	0.10–4.38		1.55		0.94	
Minutes/game 2015/16	8.98–24.38		14.80		3.69	
Minutes/game 2016/17	8.70–24.19		14.47		4.03	
Dosage 2015/16	0–1285		530.10		357.90	
Dosage 2016/17	0–2345		524.42		539.48	
Total dosage	0–3535		1066		719.50	
Outcomes	Min–max		Mean		SD	
	GBG	UP	GBG	UP	GBG	UP
T1 reading (KS1)	3–21	3–24	15.01	15.36	3.76	3.83
T2 reading (HGRT)	3–52	1–53	32.49	33.05	10.31	10.41
T3 reading (HGRT)	1–53	4–53	37.26	37.78	9.96	9.46
Demographics	School			Student		
	Overall	GBG	UP	Overall	GBG	UP
Size—number of pupils on roll	306.9	298.2	315.4	–	–	–
Sex—proportion of male students	–	–	–	52.6	50.4	54.9
FSM—proportion of pupils eligible for FSM	26.0	27.6	24.5	24.8	27.4	22.8
EAL—proportion of pupils speaking EAL	22.6	22	23.2	27.3	26.2	31
Ethnic minority—proportion of ethnic minority pupils	32.9	32.4	33.3	33.5	32.8	34.2
SEND—proportion of pupils with SEND	19.5	20.9	18.2	20.3	23.1	17.6

Schools that formally ceased implementation were given a dosage of zero from the point at which they ceased playing

*KS1* Key Stage 1, *HGRT* The Hodder Group Reading Test

^†^Game delivery delayed at T1 due to initial training and scoreboard development

### Sample Size

With an intra-cluster correlation coefficient of 0.08 for the outcome measure at baseline (Hedges and Hedberg 2007), an average cluster size of 40, standard power and alpha thresholds of 0.80 and 0.05, respectively, and a pre-post correlation (R^2^_L2_) of 0.75, the minimum detectable effect size (MDES) for an ITT analysis was determined to be 0.13.

### Intervention

The GBG is an “interdependent group-oriented contingency management procedure” (Tingstrom et al. 2006, p. 225). Core components are (1) *classroom rules*, (2) *team membership*, (3) *monitoring behavior*, and (4) *positive reinforcement*. While playing the game, students are divided into teams of up to seven. These are typically gender-balanced and heterogeneous in behavior and academic ability. Teams attempt to win GBG in order to access certain rewards or privileges. To do so, they need to have four or fewer infractions on the scoreboard at the end of the game. During game play, teachers records any infractions against one of four rules: (1) *we will work quietly*,^1^ (2) *we will be polite to others*, (3) *we will get out of our seats with permission*, and (4) *we will follow directions* (Kellam et al. 2011). It is recommended that initially the game be played three times a week, for 10 min each time, increasing over the year to every day for up to 30 min. It should also be played at varying points throughout the day, during an assortment of lessons and activities. The game is designed to be integrated into the existing curriculum.

Teachers in GBG schools attended two training days prior to implementation, with a further day of top-up training later in the academic year. Trained coaches visited teachers approximately once per month throughout the trial to support their implementation efforts (e.g., modeling of game sessions, observation and feedback; Ashworth et al. 2018).

### Implementation

In the main trial, data were collected pertaining to multiple dimensions of implementation (Humphrey et al. 2018). In brief, independent observations conducted by the research team suggested that levels of implementation *fidelity* (2015/16: 69.79%; 2016/17: 70.11%), *reach* (2015/16: 95.26%; 2016/17: 95.98%), and *participant responsiveness* (2015/16: 74.51%; 2016/17: 69.07%) were high. Thus, most of the prescribed procedures associated with the game were followed, almost all children in a given class were present when it was played, and they responded favorably (for example, correcting their behavior following an infraction). However, in the current study, we focus on *dosage*, because of the need for a single compliance marker, and the fact that the primary motivation for the CACE parameter is to determine treatment effects following *receipt* of an intervention (as opposed to the *offer* of an intervention, as in ITT estimation). Dosage is arguably the optimal indicator of this, as the unit of measurement is the amount of the intervention delivered.

An online GBG scoreboard was developed as part of the trial for teachers to record details of games, including infractions, and duration and frequency of gameplay. This minimized data burden for teachers (as they were not required to complete additional logs) and guarded against the bias associated with self-report methods that are typically utilized in implementation research (Domitrovich et al. 2010). Computer-based data collection has also been found to be more accurate in the GBG than the typically used hand-collected data procedure (Elswick et al. 2016).

In terms of frequency (Table 1), the GBG was implemented twice per week in the first year, but this reduced somewhat in the second year. The average game duration in both years was approximately 15 min. Thus, while average *duration* was well within the range of the only other GBG trials that have reported dosage data (e.g., 10 min per game in Domitrovich et al. 2015), the *frequency* of game play was lower (Domitrovich et al. 2015; Hagermoser Sanetti and Fallon 2011; Kellam et al. 1998; Pas et al. 2015). Additionally, three schools formally ceased implementation by the end of the first year, with a further six discontinuing by the end of the second year (though their dosage data are included in the above estimates). However, as Becker et al. (2013) noted regarding the GBG, “it is unknown whether a certain dosage… is necessary or sufficient to bring about student gains…as is typical of most interventions, these benchmarks have not been empirically validated” (p.221). Accordingly, the scoreboard data were used to ascertain *cumulative intervention intensity* (as in Warren et al. 2007), with dosage treated as a continuous variable representing total number of minutes’ exposure across the 2 years.

### Measures

#### Reading

Key Stage 1 (KS1) teacher assessment scores (specifically, KS1 National Curriculum reading point score: KS1_READPOINTS variable) were utilized at pre-test (referred to as T1). These data are collected in England when children are six-seven and are predictive of future academic performance (Humphrey et al. 2015). Scores were extracted from the National Pupil Database (NPD). Higher scores indicate greater reading attainment.

The Hodder Group Reading Test (HGRT; test sheet 2A) was utilized at post-test (referred to as T2) and 1-year post-intervention follow-up (T3). It was administered in a whole class context by the research team over a period of 30 min in the final term of the second year of the trial (April–July 2017) and 12 months later. The HGRT has been standardized on over 13,000 children (α = 0.95; Devine et al. 2013) and reliably measures reading ability between seven and 16 years. Higher scores indicate greater reading attainment.

#### Covariates

Disruptive behavior and concentration problems were measured using the checklist version of the Teacher Observation of Classroom Adaptation (TOCA-C; Koth et al. 2009). Data on students’ gender, FSM eligibility, and SEND status were extracted from the NPD. School-level data (size, FSM, EAL, and student absence percentages) were obtained from the Schools Information Service (formerly known as Edubase).

### Analytic Strategy

#### CACE Overview and Assumptions

For a more detailed discussion of the application of CACE to school-based intervention research, the reader is referred to Peugh et al. (2017); here, we provide only essential information. The overall aim of CACE modeling is to estimate intervention effects while accounting for compliance to the intervention. Dealing with the missing compliance data (unknown status) for the control group is challenging, given that they never received the intervention (Jo 2002a; Jo and Muthén 2001). To overcome this, CACE models are estimated probabilistically as structural equation mixture models using a discrete latent variable. This allows the identification of those in the control group who would have complied with the intervention *had they been randomized to receive it*. Potential compliers in the control group are therefore identified through their missing data, the compliance data that are available for the intervention group, and the response distribution information of the sample. The comparison of compliers from the intervention group to *potential compliers* from the control group thus becomes possible (Peugh et al. 2017).

Causal inference in CACE estimation relies on the assumption of ignorable treatment assignment (random assignment); in other words, participants were given the possibility to be exposed to either condition (Holland 1988). In addition, stable unit treatment value (SUTVA) assumes that the potential outcome of each individual is unrelated to the treatment assignment of other individuals (i.e., there was no contamination). Monotonicity assumes that none of the sample do the opposite of what they were assigned to do (in CACE terms, there are no “defiers”). Alongside this, CACE assumes that individuals in the control group did not receive the intervention (that is, there are no “always-takers”). Finally, the exclusion restriction assumption implies that the treatment effect is zero for those who did not participate (never-takers; see Angrist et al. 1996).

#### Compliance

Following the framework suggested by Angrist et al. (1996), a binary indicator of compliance (0 = non-compliers; 1 = compliers) is required in the intervention group for the identification of the latent compliance variable. In the absence of a verified cutoff, sensitivity analyses were conducted, where compliance was defined in one of two ways (as in Berg et al. 2017): (1) classrooms that fell above the 50th percentile (1030 min) were deemed to be *moderate* compliers (*n*_student_ = 672, 43.1%); (2) classrooms that fell above the 75th percentile (1348 min) were deemed to be *high* compliers (*n*_student_ = 333, 21.3%). It is important to note that while dosage data was collected at the teacher-level, it was necessary to disaggregate this information to the student-level, as information on the class membership for the control schools was not available. This is typical of educational research and modeling compliance on the lower-level was shown to work well in multilevel CACE (Jo et al. 2008).

### Statistical Analysis

Of participants in the sample, 18.3% had incomplete data, in cases where they had left the school or were absent on the day of testing at T2. This increased at T3 to 24.4%. There was no attrition at the school level; the schools that discontinued implementation still complied with data collection protocols. Missing value analysis was conducted through binary logistic regression to identify the variables that predicted partially observed data, and data were found to be missing at random (MAR; Rubin 1976). Models were estimated in Mplus 8.2 with full information maximum likelihood with robust standard errors (MLR) under the assumption of data MAR. Models were fitted using a multilevel framework, with level 1 representing students (*N* = 3084) and level 2 their schools (*N* = 77 with average cluster size = 40.05). Where a statistically significant intervention effect was observed, an effect size comparable to Cohen (1992) was calculated using the following formula Δ = *β*/*σ*_*e*_, where *β* represents the binary treatment standardized beta effect and *σ*_*e*_ indicates the student-level standard deviation of the outcome variable (Tymms 2004).

#### ITT Analysis

Two-level multiple linear regression was employed for ITT models, with treatment assignment (1 = UP; 2 = GBG), along with all student- and school-level covariates regressed on the outcome variable at both time points. Analyses assumed the intervention group were fully compliant to the intervention (Gupta 2011; Peugh et al. 2017).

#### CACE Analysis

CACE was estimated through multilevel mixture models, using MLR estimation and expectation maximization algorithm, which enables the estimation of the unknown compliance of the control group (Muthén and Muthén 2017). High starting values were used (4000 1000), and the optimization history of the models was inspected to ensure that the best loglikelihood was replicated. For the estimation of the CACE models we were confident that the above assumptions were met. Therefore, only two sub-populations were defined: compliers and ‘never-takers’ (henceforth referred to as *non-compliers*). However, meeting the exclusion restriction assumption was less likely given the arbitrary thresholds used to define compliance. For instance, students could still potentially be affected by the GBG even at lower levels of exposure (Berg et al. 2017). Although relaxing this assumption is possible with the inclusion of strong predictors of compliance (Jo 2002a), the effectiveness of this method has been less studied within multilevel CACE (Jo et al. 2008), and has received no empirical support within multilevel CACE with missing data (Jo et al. 2010). Following Panayiotou et al. (2019), we therefore assumed that intervention effects do not vary across different covariate values (i.e., additivity; Jo 2002b) and based our analysis on the inclusion of good predictors of compliance, which can substantially reduce the bias when this assumption is violated (Jo et al. 2008; Jo 2002a).

#### Covariates

Including good predictors of compliance can increase precision in estimating the latent class compliance variable and, therefore, increase power to detect CACE effects (Jo et al. 2008b). Student- (gender, FSM eligibility, SEND, baseline reading scores, concentration problems, and disruptive behaviors) and school-level (size, FSM %, EAL %, absences %) characteristics were thus added as covariates of reading scores and the latent compliance variable, as research shows that implementation can be influenced by the classroom climate, and student- and school-level characteristics (Koth et al. 2008; McIntosh et al. 2016; Pas et al. 2014; Payne and Eckert 2010). We paid particular attention to including predictors that were aligned with the behavioral focus of the GBG (Panayiotou et al. 2019 Nagengast et al. 2018). For instance, we expected that teachers would be less likely to deliver frequently in classrooms with low baseline levels of disruptive behavior.

## Results

Descriptive statistics for dosage and outcome data are presented in Table 1. ITT and CACE models are presented in Tables 2 (T2) and 3 (T3).

**Table 2:** ITT and CACE models of reading T2

		CACE *β* (SE)			
		Compliers		Non-compliers	
	ITT *β* (SE)	Moderate	High	Moderate	High
*N* (%)	3084 (100%)	1540 (50%)	815 (26%)	1544 (50%)	2269 (74%)
Student level (*R*^2^)	0.55***	0.57***	0.57***	0.63***	0.61***
Baseline (T1)	0.77 (0.01)***	0.75 (0.10)***	0.74 (0.04)***	0.79 (0.19)***	0.78 (0.02)***
Concentration problems	− 0.06 (0.02)**	− 0.05 (0.07)	− 0.07 (0.08)	− 0.08 (0.66)	− 0.07 (0.03)*
Disruptive behavior	− 0.02 (0.02)	− 0.05 (0.20)	− 0.11 (0.07)	0.01 (0.54)	0.01 (0.69)
Gender (1 = male; 2 = female)	0.01 (0.01)	0.03 (0.09)	0.03 (0.05)	− 0.01 (0.15)	0.01 (0.02)
Free school meals (0 = no; 1 = yes)	− 0.01 (0.02)	− 0.02 (0.26)	− 0.09 (0.04)*	− 0.00 (0.05)	0.01 (0.02)
Special educational needs and disabilities (0 = no; 1 = yes)	− 0.04 (0.02)*	− 0.04 (0.56)	0.01 (0.06)	− 0.03 (0.45)	− 0.05 (0.02)*
School level (*R*^2^)	0.45***	0.61	0.66***	0.66	0.55***
Trial (1 = UP; 2 = GBG)	*0.26 (0.20)*	*0.01 (1.56)*	*0.20 (0.29)*		
Free school meals %	− 0.50 (0.12)***	− 0.85 (1.75)	− 0.39 (0.18)*	0.02 (0.73)	− 0.30 (0.14)*
School size	0.04 (0.10)	− 0.04 (0.23)	0.01 (0.23)	0.31 (2.55)	0.88 (0.16)***
School absences %	− 0.28 (0.12)*	0.23 (3.17)	− 0.29 (0.18)*	− 0.88 (0.40)*	0.07 (0.13)
English as additional language %	− 0.11 (0.12)	0.25 (3.06)	0.23 (0.15)	− 0.12 (1.44)	− 0.48 (0.18)**

In italics are ITT and CACE effects

*p < .05; **p < .01; ***p < .001

**Table 3:** ITT and CACE models of reading T3 (sleeper effects)

		CACE *β* (SE)			
		Compliers		Non-compliers	
	ITT *β* (SE)	Moderate	High	Moderate	High
*N* (%)	3084 (100%)	1669 (54%)	815 (26%)	1415 (46%)	2269 (74%)
Student level (*R*^2^)	0.57***	0.62***	0.52***	0.54***	0.62***
Baseline (T1)	0.75 (0.01)***	0.78 (0.02)***	0.68 (0.04)***	0.71 (0.03)***	0.78 (0.02)***
Concentration problems	− 0.09 (0.02)***	− 0.04 (0.04)	− 0.17 (0.07)**	− 0.16 (0.03)***	− 0.07 (0.03)*
Disruptive behavior	− 0.05 (0.02)*	− 0.00 (0.03)	− 0.14 (0.07)**	− 0.09 (0.03)**	− 0.01 (0.03)
Gender (1 = male; 2 = female)	0.03 (0.02)	0.01 (0.02)	0.03 (0.04)	0.05 (0.02)*	0.03 (0.02)
Free school meals (0 = no; 1 = yes)	− 0.02 (0.02)	− 0.01 (0.02)	− 0.04 (0.05)	− 0.03 (0.03)	− 0.00 (0.02)
Special educational needs and disabilities (0 = no; 1 = yes)	− 03 (0.02)	− 0.07 (0.03)*	0.06 (0.05)	0.01 (0.03)	− 0.06 (0.02)*
School level (*R*^2^)	0.46***	0.49***	0.38	0.63***	0.42***
Trial (1 = UP; 2 = GBG)	*0.18 (0.20)*	0.93 (0.35)** (Δ = 0.10)	*− 0.25 (0.84)*		
Free school meals %	− 0.44 (0.10)***	− 0.22 (0.25)	− 0.30 (0.18)	− 0.47 (0.09)***	− 0.23 (0.25)
School size	− 0.04 (0.11)	0.25 (0.30)	0.42 (0.73)	− 0.05 (0.14)	0.08 (0.16)
School absences %	− 0.34 (0.12)**	− 0.40 (0.25)	− 0.36 (0.33)	− 0.45 (0.11)***	− 0.53 (0.24)*
English as additional language %	0.06 (0.11)	0.13 (0.29)	− 0.11 (0.45)	0.19 (0.10)	0.05 (0.13)

In italics are ITT and CACE effects

*p < .05; **p < .01; ***p < .001

### ITT Analyses

After controlling for student-level and school-level covariates, there was no statistically significant effect of the GBG on children’s reading scores at T2 (*β* = 0.26, *p* > .05) or T3 (*β* = 0.18, *p* > .05). It is worth noting that the majority of the variance in reading was predicted by baseline scores (*β* = 0.77 at T2, *β* = 0.75 at T3, both *p* < .001).

### CACE Analyses

Models were shown to have classes with no less than 1% total count, and with high posterior probabilities (> 90%) and acceptable entropy values (0.73–0.78), indicating appropriate and easily distinguished classes (Grimm et al. 2017; Jung and Wickrama 2008). At T2, there were no statistically significant effects for moderate (*β* = 0.01, *p* > .05) or high compliance (*β* = 0.20, *p* > .05). However, at T3, a small but statistically significant intervention effect was observed in the moderate compliance model (*β* = 0.93; Δ = 0.10, *p* < .01). No effect was observed in the high compliance model (*β* = − 0.25, *p* > .05).

#### Predictors of Compliance

Disruptive behaviors were shown to predict moderate compliance at T3 (*b* = 0.56, *p* < .01; odds ratio [OR] = 1.74) and high compliance at both time points (T2 *b* = 0.95, *p* < .01; OR = 2.60; T3 *b* = 0.99, *p* < .01; OR = 2.69). Student-level FSM eligibility was also a statistically significant predictor of high compliance at both time points (T2 *b* = 0.42, *p* < .05; OR = 1.52; T3 *b* = 0.51, *p* < .05; OR = 1.67).

## Discussion

The results of this RCT demonstrate that the GBG had no main effect on students’ reading attainment, either immediately at post-test, or at 1-year post-intervention follow-up. Our CACE analyses revealed no moderating effect of dosage at post-test; thus, the lack of main effect was not the result of insufficient intervention exposure. However, these analyses did reveal a small but statistically significant intervention effect among moderate compliers at 1-year follow-up. In other words, the GBG produced a sleeper effect on reading attainment when teachers played the game for between 1030 and 1347 min over 2 years.

These findings can be considered robust and credible for several reasons. First, the use of a cluster RCT minimized the possibility of diffusion or contamination effects (Campbell et al. 2001) and the violation of CACE assumptions (e.g., SUTVA; Jo et al. 2008). Second, the trial arms were well balanced at school and student levels. Third, the study was well powered. Fourth, while there was student-level attrition over time, this was within acceptable limits (Dumville et al. 2006) and was addressed via FIML. Fifth, CACE estimation enabled us to robustly determine the extent to which any ITT effects changed once dosage was taken into account. Finally, the GBG was tested in isolation, removing the confounding influence of other interventions evident in some earlier research.

### Null Results at Post-test

The mixed evidence regarding *immediate* effects of the GBG on attainment made it unclear if reading scores would improve at post-test. Indeed, our findings are consistent with those of Dolan et al. (1993) and Dion et al. (2011) in this regard. The current study extends understanding of this lack of effect by demonstrating that it is not underpinned by implementation variability (specifically, dosage), as there were also no CACE effects.

It is possible that these findings reflect a lack of cultural transferability of the GBG. While it has been found to be effective in other countries, it was adapted to suit the school culture in the Netherlands (van Lier et al. 2004), France (Dion et al. 2011) and Spain (Ruiz-Olivares et al. 2010). However, in England, the GBG was implemented in its original format. Although it was piloted, qualitative data did indicate that teachers had several concerns including the time required to implement, and the inflexibility of certain procedural elements (Chan et al. 2012). These issues were also raised in the process evaluation component of the current study (Humphrey et al. 2018), and may have impacted upon the implementation of the game, thus diluting its effects (Wigelsworth et al. 2016). It is also worth considering the sizeable effect of baseline reading scores (*β* = 0.74–0.79). This adds to a long line of work (e.g., Ashworth et al. 2019) demonstrating similar stability over time and might indicate that the GBG is not able to produce meaningful change once prior attainment is taken into account. However, another plausible explanation is that effects of this intervention on attainment simply take longer to become evident—a point to which we now turn.

### Intervention Effect Among Compliers at 1-Year Follow-Up

Similarly to the analyses conducted immediately post-test, there was no effect found at the ITT level on students’ reading point scores at the 1-year follow-up stage. One immediate possibility is that this analysis was conducted too soon and sleeper effects may still emerge. This is in some ways consistent with the logic model, where effects on attainment are only hypothesized to emerge in the short or medium term (Chan et al. 2012; though exactly how long this refers to is not specified). It is perhaps noteworthy that Kellam et al. (1994) found effects of the GBG on attainment at 6-year follow-up.

However, our CACE analysis revealed a small but significant intervention effect among moderate compliers at the 1-year follow-up stage. To the best of the authors’ knowledge, ours is the first study to examine the longer-term effects of a school-based intervention while accounting for implementation variability. However, some have identified similar results at the ITT level, whereby significant preventive effects only emerge after some delay (Greenberg and Abenavoli 2017). For instance, studies of the Promoting Alternative Thinking Strategies curriculum identified significant reductions in levels of aggression at later follow-up that were not evident at immediate post-test (e.g., Malti et al. 2011). Greenberg and Abenavoli (2017) argue that not all preventive effects are immediate as it takes time for changes in the intervention group population to consolidate, for small but key changes to snowball, and for the control group population to show symptoms of the issues that are the focus of prevention. The findings of the current study add another layer of complexity, suggesting that the emergence of sleeper effects can be contingent upon implementation variability during the intervention period. Our other CACE analyses indicate that the sleeper effect observed here may be mediated by compliance effects on disruptive behavior at post-test (Humphrey et al., under review); this will be formally examined in a future paper.

While the effect size identified among compliers (.10) is small when using standard thresholds, Durlak (2009) recommended that researchers, “do not reflexively resort to Cohen’s (1988) conventions” (p.923); instead, they should consider the practical or clinical value of an effect in context, and with reference to relevant prior research (Hill et al. 2008). In practical terms, the effect size identified here translates to an additional two months’ academic progress (EEF 2018). Considering that the primary focus of the GBG is behavior management, this is noteworthy, and is comparable with some meta-analytic findings for similar interventions. For example, Korpershoek et al. (2016) found a mean academic outcome effect size of 0.11 for single component behavior management programs.

However, no significant intervention effect among high compliers was found. While this is inconsistent with existing research (Berg et al. 2017; O’Connell et al. 2009), it may well be due to the smaller sample size of high compliers (*n* = 333, 21.3%), and/or the computational demand arising from the use of FIML within multilevel mixture modeling (Panayiotou et al. 2019). To test this, we ran post hoc single-level models accounting for clustering through Type = Complex, although results were unchanged. Alternatively, it may indicate an optimal dosage level of between 1030 and 1347 min over 2 years to trigger later academic progress. As previously noted, GBG dosage benchmarks have not been empirically validated, and so the levels required to bring about student gains is unknown (Becker et al. 2013). Another possibility is that teachers implementing with the highest dosage levels were those faced with high levels of need that perhaps exceeded the capacity of the GBG, as a universal intervention, to produce meaningful change. The fact that baseline disruptive behavior and FSM eligibility predicted high compliance supports this proposition.

### Implications and Future Directions

Our findings suggest that the GBG in isolation *can* lead to significant gains in children’s reading attainment, *providing* it is implemented with sufficient dosage *and* that benefits are not expected immediately. This highlights the importance of assessing trial outcomes at multiple time points, particularly when said outcomes are considered to be distal. As Greenberg and Abenevoli (2017) note, “when posttest-only studies conclude there are no impacts, such results are at best inconclusive” (p. 56–57). They recommend that for a complete evaluation of an intervention, multiple data collection points over extended periods of time should be included. The findings from the present study support this argument, but this is not currently the norm in the field. For example, only 15% of studies in Durlak et al.’s (2011) meta-analysis included > 6 months post-intervention follow-up.

Most universal school-based interventions are tested using an ITT approach (Berg et al. 2017). However, this can result in biased and untrustworthy findings, as the effects of non-compliance are not taken into account (Jo et al. 2008; Jo and Muthén 2001; O’Connell et al. 2009). The present study emphasizes the importance of collecting robust implementation data in trials, in order to ensure that ITT can be supplemented by CACE. However, while our findings demonstrate the *potential* effects of the GBG, this also comes with a caveat. Implementation was highly variable even though considerable support and resources were provided (e.g., external coaching support). In “real world” conditions, where such support and resources are less likely to be available, this variability will increase (Gottfredson et al. 2015). Thus, ITT analyses may represent the *likely* effects of the GBG.

Finally, we highlight the importance of incorporating appropriate predictors of compliance that are in line with the focus of the intervention (Nagengast et al. 2018; Stuart et al. 2008). For example, disruptive behavior was a significant predictor of compliance, and is also a focus in the program logic model as an intended outcome (Chan et al. 2012).

### Limitations

In terms of our sample, trial schools were larger than average, with higher rates of students with an SEND, eligible for FSM, and speaking EAL (DfE 2015). Furthermore, schools participating in the study were likely those where there was a greater perceived need for an intervention targeting behavior. As such, the current study sample may not have been fully representative of schools and students in England. In terms of our CACE framework, the exclusion restriction assumption may be at odds with the partial compliance observed in school-based interventions, though this was addressed in part via our sensitivity analyses and inclusion of good predictors of compliance. Also, as CACE requires a single compliance indicator, we were unable to include other potentially important implementation dimensions (e.g., procedural fidelity). In addition, due to the unknown classroom level information for the control schools, classroom was not modeled as a level in our multilevel CACE models. We therefore missed the opportunity to model teacher characteristics as potentially strong predictors of compliance. Finally, the sample division that is inherent to CACE clearly has consequences with respect to power, particularly in the high compliance models (Jo 2002c).

## Conclusions

The present study is the first RCT first of the GBG in England. It provides a comprehensive and rigorous examination of its impact on reading attainment, both immediately and at 1-year post-intervention follow-up, while robustly accounting for the moderating effect of implementation variability via CACE. We conclude that the GBG can produce measurable improvements in reading attainment, but these effects may take time to become apparent, and are contingent upon an optimal dosage range being met.

## Electronic supplementary material

ESM 1 (DOCX 36 kb)

## Footnotes

1 Adherence to “quietly” is defined as working at a “voice level” set by the teacher that is deemed to be appropriate for a particular activity.
